# Using Concept Mapping to Develop a Strategy for Self-Management Support for Underserved Populations Living With Chronic Conditions, British Columbia, August 2013–June 2014

**DOI:** 10.5888/pcd12.150183

**Published:** 2015-10-08

**Authors:** Susan L. Mills, Kim Bergeron, Guillermina Pérez

**Affiliations:** Author Affiliations: Kim Bergeron, School of Kinesiology and Health Studies, Queen’s University, Kingston, Ontario; Guillermina Pérez, School of Population and Public Health, University of British Columbia, Vancouver, British Columbia.

## Abstract

**Introduction:**

Self-management support (SMS) is an essential component of public health approaches to chronic conditions. Given increasing concerns about health equity, the needs of diverse populations must be considered. This study examined potential solutions for addressing the gaps in self-management support initiatives for underserved populations.

**Methods:**

Stakeholders representing government, nongovernment organizations, Aboriginal communities, health authorities, medical practices, and research institutions generated, sorted, and rated ideas on what could be done to improve self-management support for underserved populations. Concept mapping was used to facilitate the collection and organization of the data and to generate conceptual maps.

**Results:**

Participants generated 92 ideas that were sorted into 11 clusters (foster partnerships, promote integrated community care, enhance health care provider training, shift government policy, support community development, increase community education, enable client engagement, incorporate client support systems, recognize client capacity, tailor self-management support programs, and develop client skills, training, and tools) and grouped into system, community, and individual levels within a partnership framework.

**Conclusion:**

The strategy can stimulate public health dialogue and be a roadmap for developing SMS initiatives. It has the potential to address SMS and chronic condition inequities in underserved populations in several ways: 1) by targeting populations that have greater inequities, 2) by advocating for shifts in government policies that create and perpetuate inequities, 3) by promoting partnerships that may increase the number of SMS initiatives for underserved groups, and 4) by promoting training and engagement that increase the relevance, uptake, and overall effectiveness of SMS.

## Introduction

Self-management support (SMS) aims to improve health in chronically ill populations (1). SMS, an essential component of the Chronic Care Model (2), is driving the reform of chronic condition management in North America. SMS is increasingly embedded in national, system, and community policy documents on chronic condition management; is integrated into community-based care, primary care, and healthy aging (3); and is considered part of a public health strategy (4). However, SMS is largely failing to meet the needs of many disadvantaged populations living with complex needs (5). These populations are less likely to benefit from SMS because of low uptake, low attendance at clinical appointments, and high attrition rates (eg, not attending programs or treatment) (6–8), possibly because of low rates of health literacy, high rates of multiple diseases, and difficulties in access to care (1,5,9). Some populations have higher prevalence rates for chronic conditions, greater difficulties in managing their conditions, and worse health care outcomes (10,11). SMS approaches that fail to help these groups may exacerbate inequities (5,6).

An international framework identified key strategies for developing a comprehensive approach to SMS and emphasized the need to address health equity concerns (12). In Canada, few initiatives address SMS for underserved populations (3), although the need to address this health equity gap has been identified in national and international settings (1,5). To this end, a group of decision makers who have expertise in providing SMS to underserved populations living with chronic conditions and who represent government, nongovernment, research organizations, health authorities, Aboriginal communities, and medical practices were brought together to discuss how they could improve SMS for underserved populations. The purpose of this study was to facilitate knowledge exchange among these stakeholders to identify priorities and actions for improving SMS for underserved populations in British Columbia.

## Methods

Numerous methods are available for gathering and organizing data from a group of respondents (eg, Delphi technique); however, we chose concept mapping because concept maps provide a systematic way to organize and rank stakeholders’ ideas and understand how the ideas relate to each other, which is helpful in the development of a strategy (13–15). We engaged in 3 major concept-mapping activities: 1) brainstorming ideas, 2) sorting and rating ideas, and 3) analyzing and interpreting the concept maps (15). We used a website for the brainstorming activity, a face-to-face 1-day workshop for all 3 activities, an online survey, and an interactive webinar for analysis and interpretation. We used Concept Systems software, version 2013.322.11 (Concept Systems, Inc) to sort and rate ideas. A researcher (K.B.), who is a certified Concept Systems facilitator, guided the design and implementation of the concept-mapping process and facilitated the workshop. Ethics approval was obtained from the Behavioral Research Ethics Board at the University of British Columbia (BREB Number: H13–02557). This study was conducted from August 2013 through June 2014.

### Procedures

#### Recruitment

A purposeful sampling technique (16) was used to recruit participants who had experience working with diverse underserved populations (eg, racial/ethnic minorities, immigrants, refugees, low-income adults, older adults, homeless people, rural residents, Aboriginal populations) living with a range of chronic conditions prevalent in these populations (eg, HIV, diabetes, mental illness) (17–19). Eligibility criteria were that participants worked in British Columbia and had knowledge and experience in providing SMS to underserved populations living with a chronic condition. SMS was defined as the systems, policies, services, and programs that extend across health care, social sectors, and communities to support and improve the way people manage their own chronic conditions, optimize their health, and live well (20). A steering committee recommended potential participants who met the inclusion criteria, and invitations to participate in the study were sent by email. A total of 26 people participated in 1 or more phases of the study (brainstorming involved 26 participants; sorting and rating, 25; interpretation of maps, 24; postworkshop survey, 17; and webinar, 9). The sample size for each concept-mapping activity was sufficient to meet the statistical requirements for a valid and reliable result (eg, brainstorming, >20; sorting and rating, >10) (14,21).

Most participants worked with several underserved populations that lived with a range of different (and often multiple) chronic conditions. Approximately 70% of participants worked in health authorities and educational institutions, and approximately 35% had more than 10 years of experience working in the SMS field (Table 1).

**Table 1:** Characteristics of Study Participants (N = 26), British Columbia, August 2013–June 2014

Characteristics	Number
**Work place**	
Educational institution	6
Government	1
Health authority	12
Not for profit	4
Private business/for profit	1
None of the above	2
**Experience in self-management support field, y**	
<1	1
1–5	7
6–10	6
11–20	9
>20	2
Unknown	1

#### Brainstorming and idea generation

Participants who agreed to take part in the Web-based online brainstorming phase were asked to generate responses to the following question: What could be done to improve self-management support for underserved populations living with chronic conditions in British Columbia? Ideas submitted were anonymous but could be viewed by all online participants. During 2 weeks, 109 ideas were generated; ideas were then synthesized by the researchers to remove similar or duplicate ideas (15). Seventy-nine ideas were presented at the face-to-face workshop, and attendees had an opportunity to review and suggest new ideas not included in the synthesized list. An additional 13 unique ideas were added for a final set of 92 ideas.

#### Structuring the ideas by sorting and rating 

The 92 ideas were entered into Concept Systems software. The software randomized the ideas to create sorting and rating cards. Participants were provided with a set of cards (1 idea per card) and were asked to organize them into groups based on perceived similarity in meaning and to give each group of cards a name that reflected the theme the ideas represented. Participants were also provided rating worksheets that listed the 92 ideas alongside a 5-point Likert scale (1= relatively unimportant/not feasible, 2 = somewhat important/feasible, 3 = moderately important/feasible, 4 = very important/feasible, 5 = extremely important/feasible). Participants were instructed to scan the entire list of ideas and rate each idea on the scale by considering the importance of the idea relative to the other ideas generated and the feasibility of implementing the idea in the next 3 years. Although a range of criteria could have been used, importance and feasibility were proposed by the research team and endorsed by participants because of the perceived relevance of these criteria to current policy and practice environments and their well-documented use in concept mapping studies for planning purposes (13,14). 

### Data analysis and interpretation of the concept maps

Four sequential activities were used to analyze and interpret the results: 1) an initial face-to-face workshop to interpret selected maps, 2) postworkshop calculation and interpretation of new maps, 3) online survey for validation of results and generation of potential implementation actions, and 4) interactive webinar discussion to review revised findings and survey results and to generate ideas for developing a strategy.

### Face-to-face workshop

**Figure 1:**
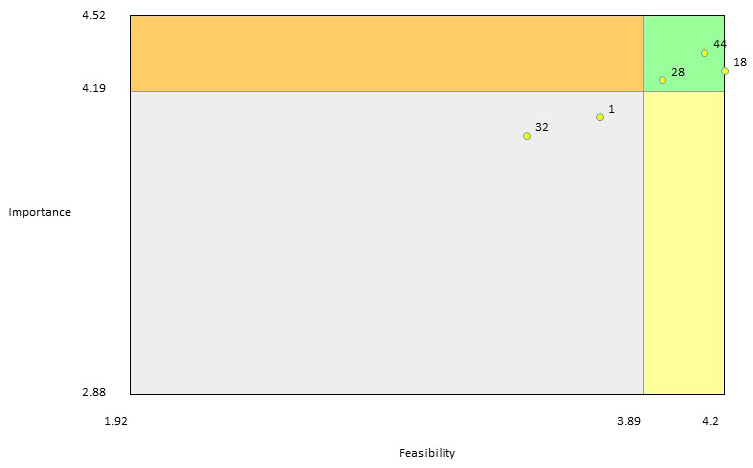
“Go zone” map for the cluster “fostering partnership” shows the average importance and feasibility rating data for the ideas included in this cluster. The right upper quadrant (green zone) represents the ideas that were rated above average on both importance and feasibility.

The sorting and rating data were analyzed by using concept mapping software, which uses multidimensional scaling analysis and hierarchical cluster analysis to depict relationships between ideas, create clusters, and generate concept maps (15). The first map generated — a point cluster map — showed a visual arrangement of the 92 ideas plotted on an *x–y *graph (Figure 1). The closer the points were to each other, the more often participants sorted ideas together (15). A stress value, which reflects how often the ideas were sorted together, was calculated as 0.34, which is an acceptable value because it falls between the ideal range of 0.28 and 0.39 (14). We then used the software to create cluster maps that displayed the ideas as 2-dimensional polygons on the basis of how conceptually similar or dissimilar the ideas were to each other (15). The distance between the ideas, rather than the exact location of the ideas on the map, illustrates the degree of similarity between ideas. The software generated numerous cluster maps, each of which displayed a different number of clusters (from 23 clusters to 5 clusters). Recognizing that we would have to follow the recommended process for cluster selection (15) after the workshop, we shared a 10-cluster solution with participants because we felt this provided a reasonable number of clusters to generate preliminary discussion. Working in small groups, participants reviewed how the ideas were grouped and then reviewed the cluster names to see if they represented the dominant themes for each set of ideas. During this process, participants struggled to find a common theme among the ideas in each cluster. They felt that a 10-cluster solution was not likely the best solution for developing an SMS strategy and that many of the software-generated cluster names did not reflect the major ideas expressed in each group. Participants recommended that further cluster analysis be conducted and cluster names reviewed.

### Postworkshop analysis

In a postworkshop analysis, the research team worked to find a cluster solution in which the ideas in each cluster were cohesive and represented a common theme. The concept mapping software program filter was used to eliminate spurious relationships between ideas and to reduce the “noise” in the data to provide groupings with stronger conceptual coherence (22). We applied a filter to the data to create cluster solutions based on statements sorted together by 3 or more participants. We systematically examined the ideas that merged together as clusters were reduced from 23 to 7. We used quantitative data, specifically, the bridging values of cluster solutions that were computed by the software program. The bridging value of a cluster was the average bridging value for each statement in the cluster. Clusters with low values were usually more cohesive and easier to understand. A bridging value for a statement was a measure of whether it was sorted with others that were close to it on the map (eg, a lower bridging value means that statements were more often sorted together) (15). We also used qualitative processes to interpret whether the ideas in the clusters formed coherent conceptual themes as we moved across each cluster solution (15). Participants agreed on 11 clusters representing strategic directions. We calculated the “go zone” (an *x–y* graph) for each cluster to show the bivariate plot of the average importance and feasibility rating data for each idea (15) 

### Online survey

Participants were invited to respond to an online survey that asked them to review the 11 cluster names and indicate their agreement (yes/no). If there was no agreement, participants had the opportunity to provide comments and suggest alternative names. Agreement was 71% to 83% for 10 of 11 cluster names among the 17 respondents. Participants were also invited to provide suggestions on how to implement the 40 ideas that were rated highly important and feasible.

### Facilitated interactive webinar discussion

The research team held an online webinar discussion with 9 participants to review the maps describing the 11-cluster solution and the online survey results. Final decisions were made on cluster names, prioritization of clusters, and the conceptual grouping of clusters..

## Results

Postworkshop analysis participants agreed that the 11-cluster solution provided a meaningful foundation for identifying strategies (Table 2) (Figure 1) (15). The 11 clusters (strategic directions) were foster partnerships, shift government policy, promote integrated care reform, enhance health care provider training, increase community education, enable client engagement, support community development, incorporate client support systems, recognize client capacity, develop client skills, training, and tools, and tailor SMS programs.

**Table 2:** Recommendations, Strategy for Self-Management Support (SMS) for Underserved Populations Living with Chronic Conditions: 40 Recommended Actions in 11 Strategic Directions Organized by Intervention Level, British Columbia, August 2013–June 2014

Intervention Level	Strategic Direction	Recommended Actions [No. of Ideas^a^]
System: action implemented by government, health care systems, nongovernment organizations	Foster partnerships	Build on existing programs that are working for underserved populations [44]
		Partner with agencies that have a successful track record of outreach to underserved populations to ensure that they know about, prioritize, and are able to participate in SMS programs [18]
		Deliver community-based chronic disease management programs that provide SMS in partnership with community organizations [28]
	Shift government policy	Involve underserved populations in decision making on SMS initiatives with the British Columbia Ministry of Health and Health Authorities [8]
		Advocate for politicians to implement policies to provide more social infrastructure and financial support for underserved populations to address issues of income inequality and income disparity [12]
	Promote integrated care reform	Promote interprofessional collaboration and integrated care to manage the complexity of chronic disease management and to provide SMS [46]
		Develop collaboration with educational institutions and embed SMS in their curriculum [91]
		Create a full community-based primary health care system that incorporates a full SMS model (multiple chronic diseases) and social determinants of health with a focus in TRIPLE AIM (improve population health, improve patient and provider experience at a sustainable cost) [83]
		Create processes that make transitioning both in and out of service easier and barrier-free [37]
		Involve the health care system and wider community in the expanded chronic disease models to provide the resources and infrastructure to enable SMS in underserved populations [77]
		Create an integrated system-wide approach to chronic disease management that includes SMS as one of its mandates [61]
	Enhance health care provider training	Provide health care–provider training in health literacy, cultural competency, and safety to ensure SMS initiatives are client friendly, culturally appropriate, language specific, and tailored to the literacy level and readiness of client [27]
		Ensure SMS is a routine part of regular office visits for chronic disease management [3]
		Focus services on wellness as well as illness management [13]
		Train staff in SMS skills (eg, health coaching, mindfulness, motivational interviewing, goal setting, problem solving, action planning with clients) as part of basic health care professional training to use in all SMS programs and services [54]
Community: action addressed by communities and related organizations	Increase community education	Increase awareness of Aboriginal people and history of colonization (residential school) [80]
		Increase awareness of and reduce the stigma of mental health conditions [63]
		Ensure a shared understanding with clients of core concepts of self-management and support to be able to know when success has been achieved [84]
	Enable client engagement	Engage underserved populations in identifying barriers to self-management and in finding potential solutions [41]
		Promote healthy communities where citizens are encouraged and invited to contribute [85]
		Work with multiple stakeholders to ensure that SMS programs are accessible (ie, sociocultural alignment with the target population, affordable or free, and in places where they feel comfortable that are easily reached on foot or with public transit) [51]
	Support community development	Support local communities to create their own programs [81]
		Engage underserved populations with lived experience of chronic conditions as equal partners in creating all SMS policy and program/systems planning, and research processes [68]
Individual: action directed toward clients.	Incorporate client support systems	Build on client support systems and involve families and caregivers in SMS [40]
		Incorporate more peer leaders and peer experts into community-based primary health care [29]
		Provide accessible venues for groups of individuals with similar chronic conditions to meet and provide support to each other [2]
		Provide coaching (telephone or face to face) to support people in their wellness [56]
		Train health coaches to provide ongoing support to help clients manage their disease, navigate the health care system, and access resources [49]
	Recognize client capacity	Develop SMS that takes into account varying literacy and health literacy skills in underserved populations (eg, individuals may not be literate in their first language and may require alternative strategies and forms of communication) [67]
		Use translators that are linguistically and culturally aligned with clients [74]
		Build on the personal agency of clients given, for example, their language or literacy skills [71]
	Develop client skills, training, and tools	Use experiential learning approaches to teach skills clients need to self-manage (eg, engage them in cooking healthy meals that are ethnoculturally appropriate rather than showing them the nutrition pyramid) [70]
		Develop appropriate, accessible, evidence-based resources and tools that clients can use to help manage their chronic conditions [75]
		Offer clients skills training to effectively self-manage (eg, information and education about the disease, strategies to stall progress and prevent complications, skills to manage the disease on day-to-day, problem-solving, coping techniques) [16]
		Consider differences within underserved populations when SMS facilitators are implementing programs (eg, for immigrants, socioeconomic status, urban or rural origins, and time since immigration influence both cultural expressions and language skills, which in turn affect understanding and uptake of SMS) [6]
		Develop teaching and learning models for clients to develop skills to effectively engage with health professionals in shared decision making [21]
		Ensure self-reflection tools in SMS to ensure measurements are in place for progress [86]
	Tailor SMS programs	When creating SMS programs for physical conditions include content on mental health issues [10]
		Use a holistic approach to SMS initiatives that considers physical, cultural, lifestyle, and spiritual needs of underserved populations [52]
		Develop materials and programs for health literacy (including e-health literacy, computer-based health information) that can be used by health care professionals [5]

Abbreviation: SMS, self-management support.

**Figure 2:**
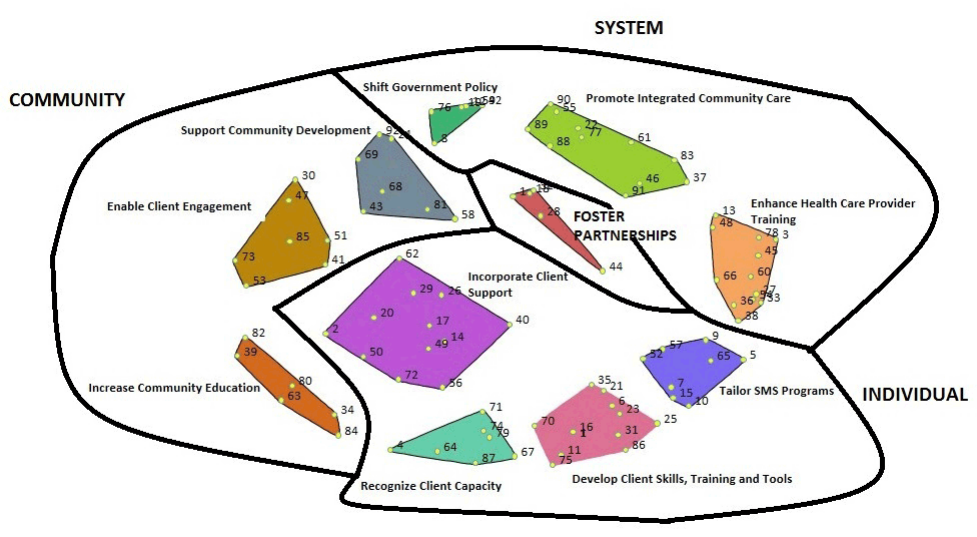
The final 11-cluster solution of the 92 statements generated, grouped by 4 conceptual groupings: 1) fostering partnerships, 2) systems level system (actions implemented by governments, health care systems, nongovernment organizations), 3) community (actions addressed by communities and related organizations), and 4) individual (actions directed toward clients). The black lines represent the conceptual groupings of the 11 clusters.

a The numbers in brackets represent the original number of the ideas presented in Figure 2.

On the basis of the positioning of the clusters on the go-zone maps, it was decided that the cluster strategies should be considered at 3 interrelated levels: 1) system (actions implemented by governments, health care systems, nongovernment organizations), 2) community (actions addressed by communities and related organizations), and 3) individual (actions directed toward clients); we organized the 11 clusters in relation to the levels identified (Figure 2). Participants considered the “foster partnerships” cluster (centrally located on the map) the foundation for the other 3 groups because partnerships were inherent in many of the ideas in the other clusters. This conceptual structure formed the foundation for the SMS strategy for underserved populations. The collective results of the go-zone maps for each cluster showed that 40 of the 92 ideas were located in the upper right quadrants and represented the ideas that had above-average ratings for importance and feasibility. Participants agreed that these 40 ideas should form the recommended actions in the strategy. The resulting strategy comprised the 11clusters (11 strategic directions) and 40 recommended actions for improving SMS for underserved populations in British Columbia (Table 2). 

## Discussion

Twenty-six stakeholders from policy, practice, and research sectors identified a strategy with 11 strategic directions and 40 recommended actions that address the question of what could be done to improve SMS for underserved populations living with chronic conditions in British Columbia. Results showed that efforts need to be directed at 3 interrelated levels: 1) system; 2) community; and 3) individual. This understanding aligns with a systems-oriented approach to public health (23) and with the knowledge that initiatives interacting across multiple sectors are required to improve health outcomes for underserved populations (24). The strategy does not provide details on how to implement the strategic directions and recommended actions. The development of implementation plans requires further local and context-specific conversations with underserved populations living in different regions on how best to advance the strategic directions and recommended actions.

The SMS strategy can address health inequities in SMS and chronic condition outcomes in underserved populations in numerous ways. First, it targets populations that have greater inequities, such as racial/ethnic minorities, immigrants, refugees, low-income adults older adults, homeless people, rural residents, and Aboriginal populations. Second, the strategy contains recommended actions to help change government policies (eg, income policies) that create inequities in chronic conditions in underserved populations (11). Third, the partnership framework promotes collaborations between governments, health authorities, health care organizations, and community agencies that could increase the number and range of SMS-related initiatives for underserved groups by sharing and leveraging skills, capacities, and resources (25). Finally, the key strategies on enhancing health care professional training on issues specific to SMS in underserved populations (eg, cultural competency, health literacy), engaging underserved populations in identifying barriers and generating solutions, and gearing SMS programs to client capacity all work toward increasing more effective implementation and better health outcomes for underserved populations living with chronic conditions (26).

Concept mapping was a good technique to develop a strategic plan because of the participatory process, which enabled stakeholders working at different levels in the system to have equal opportunity to propose ideas. It enabled decision makers to participate directly in research to generate knowledge relevant to their work domains (13) and provided a relatively quick way to generate a plan among participants in different geographical locations. Because concept mapping uses a range of data collection methods (individual and group activities) and different types of analysis (quantitative and qualitative), we were able to incorporate the benefits of each form of engagement with a mixed-method approach to address a complex topic efficiently during a relatively short time (27).

The findings of our study represent the opinions of a few stakeholders working in British Columbia, and it is likely that some perspectives were not captured in the concept mapping process nor reflected in the final strategy. Members of underserved communities living with chronic conditions were not included because of concerns about harms that might result from engaging in concept mapping without adequate training (eg, further marginalization, tokenism). A decision was made to engage consumers in future discussions of the strategy through participants’ organizations. Although we had participants from nongovernment organizations serving the health and social needs of underserved populations (eg, Vancouver Native Health Society) and Aboriginal communities (eg, Sechelt First Nation), most attendees represented organizations centered on health care rather than social services, which may have also influenced the findings.

The SMS strategy for underserved populations represents the ideas of policy, practice, and research stakeholders working in British Columbia, but the priorities and recommended actions align with SMS and health equity developments in other provinces in Canada (28) as well as in the United States, Australia, and the United Kingdom (5). As such, the SMS strategy may be relevant to regions outside British Columbia. The strategy can be used to stimulate further public health dialogue on SMS in underserved populations and as a roadmap for decision makers to help guide the development of initiatives that work toward reducing chronic condition management inequities in these groups.
